# Risk factors for surgical site infection after malignant bone tumor resection and reconstruction

**DOI:** 10.1186/s12885-019-5270-8

**Published:** 2019-01-08

**Authors:** Shinji Miwa, Toshiharu Shirai, Norio Yamamoto, Katsuhiro Hayashi, Akihiko Takeuchi, Kaoru Tada, Yoshitomo Kajino, Takashi Higuchi, Kensaku Abe, Hisaki Aiba, Yuta Taniguchi, Hiroyuki Tsuchiya

**Affiliations:** 1 Department of Orthopaedic Surgery, Kanazawa University School of Medicine, Kanazawa, 920-8641 Japan; 2 Department of Orthopaedics, Graduate School of Medical Science, Kyoto Prefectural University of Medicine, Kyoto, 602-8566 Japan

**Keywords:** Bone tumor, Surgical site infection, Risk factor, Iodine-coated implant, Multivariate analysis

## Abstract

**Background:**

Use of an implant is one of the risk factors for surgical site infection (SSI) after malignant bone tumor resection. We developed a new technique of coating titanium implant surfaces with iodine to prevent infection. In this retrospective study, we investigated the risk factors for SSI after malignant bone tumor resection and to evaluate the efficacy of iodine-coated implants for preventing SSI.

**Methods:**

Data from 302 patients with malignant bone tumors who underwent malignant bone tumor resection and reconstruction were reviewed. Univariate analyses were performed, followed by multivariate analysis to identify risk factors for SSI based on the treatment and clinical characteristics.

**Results:**

The frequency of SSI was 10.9% (33/302 tumors). Pelvic bone tumor (OR: 4.8, 95% CI: 1.8–13.4) and an operative time ≥ 5 h (OR: 3.4, 95% CI: 1.2–9.6) were independent risk factors for SSI. An iodine-coated implant significantly decreased the risk of SSI (OR: 0.3, 95% CI: 0.1–0.9).

**Conclusion:**

The present data indicate that pelvic bone tumor and long operative time are risk factors for SSI after malignant bone tumor resection and reconstruction, and that iodine coating may be a promising technique for preventing SSI.

## Background

Surgical site infection (SSI) remains one of the biggest problems associated with early failure of reconstructions with implants after bone tumor resection. Although prostheses, intramedullary nails, and plates are commonly used during reconstruction after bone tumor resection, a high infection rate after resection and reconstruction with implants has been reported [1–3]. Patients with deep infection require implant removal, irrigation, and prolonged antibiotic use to manage the surgical site.

Recently, we developed a new procedure for anodization of iodine-containing surfaces that could directly support existing titanium implants. In a basic research study, iodine-supported titanium showed good antibacterial activity and biocompatibility without cytotoxicity [4]. Since 2008, iodine-supported implants have been used in patients with infection or at high risk of infection [5, 6]. In bone tumor surgery, iodine-coated implants have been used in patients with a high risk of SSI. While determining the efficacy of iodine-coated implants in preventing SSI, the influence of several factors, such as preoperative chemotherapy and surgical site, should be considered. The objectives of this study were to determine risk factors associated with the development of SSI, and to investigate the efficacy of iodine-coated implants for preventing SSI after bone tumor resection.

## Methods

### Patients

Overall, this study included 302 patients with malignant bone tumors, who underwent tumor excision and reconstruction using plate, screw, intramedullary nail, prosthesis, and external fixation between January 1995 and July 2016. All the implants were metallic devices. There were 173 men and 129 women whose ages ranged from 4 to 92 years (mean age, 43.1 years). The diagnoses comprised of 119 metastatic tumors, 119 osteosarcomas, 26 chondrosarcomas, 19 undifferentiated pleomorphic sarcomas (malignant fibrous histiocytosis), 7 Ewing’s sarcomas, 5 adamantinomas, 3 fibrosarcomas, 1 extraskeletal myxoid chondrosarcoma, 1 malignant giant cell tumor, 1 EWSR1 rearranged sarcoma, and 1 hemangiopericytoma. Locations of the tumors included the femur (*n* = 184), tibia (*n* = 52), humerus (*n* = 36), pelvis (*n* = 24), foot (*n* = 3), radius (*n* = 2), and scapula (n = 1) (Table 1). Bone tumors located in spines were excluded from the study. In previous reports, high infection rates were reported in patients with pelvic tumor, tibial tumor, chemotherapy, radiation therapy, long operative time, or biological reconstruction [7–10]. Biological reconstruction is defined as reconstruction using bone for bony defect after resection of bone tumors. Biological reconstruction includes viable bone (iliac bone, vascularized fibula, or distraction osteogenesis), allograft, tumor-bearing autograft treated by freezing, pasteurization, autoclaving, or irradiation [11–15]. Basically, patients with at least one of these factors have been treated with iodine-coating implants since 2009, although only cases of elective surgery reconstructed by titanium implants can be treated with iodine-coated implants because the preparation of iodine-coated titanium implants requires 7 to 14 days. Sixty-six patients who underwent surgery with a high risk of SSI from 2009 to 2016 were treated with iodine-coated implants, including a prosthesis, plate, intramedullary nail, or external fixation. This study was approved by Medical Ethics Committee of Kanazawa University, and need for written informed consent was waived by the ethics committee.

**Table 1:** Characteristics of patients with uncoated and iodine-coated implants

Characteristic		Uncoated implant (*n* = 236)	Iodine-coated implant (*n* = 66)	*P* value
Age (years)		46 (range, 8–92)	32 (range, 6–85)	< 0.001
Male/Female		135/101	38/28	1.000
Diagnosis	Metastatic tumor	109	10	
	Osteosarcoma	76	43	
	Chondrosarcoma	21	5	
	MFH/UPS	16	3	
	Ewing’s sarcoma	6	1	
	Extraskeletal myxoid chondrosarcoma	1	0	
	Fibrosarcoma	2	1	
	Hemangiopericytoma	1	0	
	Malignant GCT	1	0	
	Adamantinoma	2	3	
	EWSR1 rearranged sarcoma	1	0	
Instruments	IM nail	64	0	
	Plate	44	35	
	Joint prosthesis	47	8	
	Endoprosthesis	53	20	
	Screw	6	0	
	Joint prosthesis and IM nail	1	0	
	Plate and IM nail	1	1	
	Joint prosthesis and plate	1	1	
	External fixation	19	1	
Reconstruction	Frozen autograft	91	38	
	Bone graft	10	2	
	Artificial bone and frozen autograft	0	1	
	Allograft	5	1	
	Bone cement	53	1	
	Bone graft and frozen autograft	3	0	
	Allograft and frozen autograft	3	0	
	Autoclaved bone	1	0	
Chemotherapy		152	53	0.017
Operative time (minutes)		295	327	0.152

*MFH* = malignant fibrous histiocytoma; *UPS* = undifferentiated pleomorphic sarcoma; *GCT* = giant cell tumor; *IM* = intramedullary

### Outcome measure

The incidence of SSI and its relationship with factors, including the use of iodine-coated implants, were assessed. The patient characteristics included age, site of the tumor (pelvis or other), tumor histology (primary or metastasis), recurrent tumor, fracture, chemotherapy, and radiation therapy to the surgical site. The surgery-related characteristics included surgical procedure (fixation only, prosthetic replacement, biological reconstruction without prosthetic replacement, and biological reconstruction with prosthetic replacement), the use of iodine-coated implants, additional surgery (surgeries for hematoma, fracture, nonunion, wound diastasis, breakage of implants, and perforation of intestine), and operative time. The optimal cutoff levels of age, operative time was identified by receiver operator characteristic (ROC) curve analysis. The use of an iodine-coated implant was defined as the use of a plate, intramedullary nail, or prosthesis with iodine coating. SSIs were defined using the US Centers for Disease Control classifications for SSIs [16].

### Statistical analysis

Statistical analyses were performed as described previously [17]. To assess the association between SSI after bone tumor surgeries and each factor, univariate analysis by Fisher exact test was performed. To identify the independent risk factors for SSI, multiple logistic regression analysis was performed. Any parameter with a *p*-value < 0.01 on univariate analysis and use of iodine-coated implant were included in the multiple logistic regression models. *P* value less than 0.05 was considered as statistical significance. EZR (Saitama Medical Center, Jichi Medical University) was used for statistical analyses.

## Results

### Incidence of surgical site p infection

Characteristics of patients with uncoated and iodine-coated implants were shown in Table 1. Among the study patients, the incidence of SSI was 10.9% (33/302 operations). The infection rates in patients with bone tumors in the femur, tibia, humerus, and pelvis were 4.3, 25.0, 2.8, and 41.7%, respectively (Table 2).

**Table 2:** Locations and incidence of postoperative deep infection

Locations	Number of tumors	Infection (%)
Femur	184	8 (4.3%)
Tibia	52	13 (25.0%)
Humerus	36	1 (2.8%)
Pelvis	24	10 (41.7%)
Foot	3	1 (33.3%)
Radius	2	0 (0%)
Scapula	1	0 (0%)
Total	302	33 (10.9%)

### Risk factors for surgical site infection

Univariate analyses revealed that pelvic tumor (odds ratio [OR] 7.8; confidence interval [CI] 2.8–21.5; *p* <  0.001), biological reconstruction (OR 6.8; CI 1.5–61.9; *p* = 0.004), composite use of biological reconstruction and prosthetic replacement (OR 6.1; CI 1.1–61.5; *p* = 0.019), additional surgery (OR 3.2; CI 1.3–7.4; *p* = 0.006), and operative time ≥ 5 h (OR 6.8; CI 2.7–19.1; *p* <  0.001) were significantly correlated with an increased risk of SSI (Tables 3 and 4). On the other hand, metastatic tumors and pathological fractures were correlated with a decreased risk of SSI (Table 3).

**Table 3:** Results of univariate analysis of the patient-related parameters

Factor		Number (%) of tumors with deep infection	OR	95% CI	*p* value
Age	≥40 years	15/170 (8.8%)	0.613	0.275–1.351	0.197
	< 40 years	18/132 (13.6%)			
Tumor location	Pelvis	10/24 (41.7%)	7.821	2.782–21.510	< 0.001
	Other	23/278 (8.3%)			
Metastatic tumor	Yes	7/119 (5.9%)	0.379	0.134–0.936	0.024
	No	26/183 (14.2%)			
Recurrent tumor	Yes	2/16 (12.5%)	1.174	0.124–5.501	0.690
	No	31/286 (12.9%)			
Pathological fracture	Yes	2/62 (3.2%)	0.225	0.025–0.931	0.037
	No	31/240 (12.9%)			
Chemotherapy	Yes	25/193 (13.0%)	1.634	0.679–4.371	0.326
	No	8/94 (8.5%)			
Radiation therapy	Yes	2/15 (13.3%)	1.269	0.133–6.025	0.673
	No	31/287 (10.8%)			

*OR*, odds ratio; *CI*, confidence interval

The *p* values were calculated with Fisher exact test

**Table 4:** Results of univariate analysis of the surgery-related parameters

Factor		Number (%) of tumors with deep infection	OR	95% CI	*p* value
Surgical procedure	F	2/62 (3.2%)	–	–	–
	P	3/85 (3.5%)	1.097	0.121–1.097	1
	B	20/108 (18.5%)	6.761	1.548–61.860	0.004
	B + P	8/47 (17.0%)	6.056	1.127–61.514	0.019
Iodine-coated implant	Yes	4/66 (6.1%)	0.461	0.114–1.388	0.184
	No	29/236 (12.3%)			
Operative time	≥5 h	26/121 (21.5%)	6.759	2.728–19.148	< 0.001
	< 5 h	7/181 (3.9%)			
Additional surgery	Yes	12/53 (22.6%)	3.162	1.312–7.356	0.006
	No	21/249 (8.4%)			

*F*, fixation only; *P*, prosthetic replacement; *B*, biological reconstruction without prosthetic replacement; *B + P*, biological reconstruction with prosthetic replacement; *OR*, odds ratio; *CI*, confidence interval

The p values were calculated with Fisher exact test

Pelvic bone tumor, biological reconstruction, additional surgery, long operative time, and the use of an iodine-coated implant, were included in the multiple logistic regression model. Multivariate analysis revealed that pelvic bone tumor (OR 4.9; CI 1.8–13.4; *p* = 0.002) and an operative time (OR 3.4; CI 1.2–9.6; *p* = 0.022) were independent risk factors for SSI (Table 5). The use of an iodine-coated implant was significantly associated with a decreased risk of SSI (OR 0.3; CI 0.1–0.9; *p* = 0.039).

**Table 5:** Risk factors for postoperative deep infection according to multivariate analysis

Factor	OR	95% CI	*p* value
Pelvic tumor	4.86	1.76–13.40	0.002
Operative time ≥ 5 h	3.38	1.19–9.62	0.022
Biological reconstruction	2.46	0.75–8.05	0.136
Additional surgery for complications	1.96	0.81–4.78	0.137
Use of an iodine-coated implant	0.29	0.09–0.94	0.039

*OR*, odds ratio; *CI*, confidence interval

Values were calculated by multiple logistic regression analysis

## Discussion

The introduction of chemotherapy has improved the survival rate of patients with malignant bone tumors. Furthermore, development of chemotherapy has also enabled good local control, with limb-sparing surgery being used in 90% of patients [18]. Limb sparing surgery comprises endoprosthesis, allograft, autograft, distraction osteogenesis, or artificial bone graft to reconstruct bone defects following tumor resection [19, 20]. Although limb sparing surgery is standard treatment for malignant bone tumors, there are problems with the long-term durability of the reconstruction, and some patients requires secondary amputation due to locally recurrent disease or SSI [19]. SSI requires irrigation surgery, the use of antibiotics for a long period, and delays in the treatment course, which increases mortality. In general surgery, biomaterial has been considered to be a risk factor for SSI [21]. Previous studies have reported that 9–28% of cases of infection occur after endoprosthetic reconstruction [1, 2, 22, 23]. In contrast, reconstruction without an implant is associated with a low infection rate (0.9–1.2%) [24–26]. Based on findings from previous reports, there is evidence for the use of an implant to be a strong risk factor for infection after bone tumor resection. To improve the outcomes of bone tumor surgery, new technology that prevents infection needs to be developed.

In our present study, pelvic tumor and long operative time were associated with an increased risk of infection after tumor resection and reconstruction using implants. The infection rates after resection of pelvic or tibial tumors between 15 and 43% have been reported, whereas only 4–5% of reconstructions after the resection of another part resulted in infection [7, 10, 27–30]. Therefore, the surgical site should be considered as a risk factor for SSI. The National Nosocomial Infections Surveillance (NNIS) has identified the operative time as being predictive of SSI after general surgery procedures [31]. Malignant disease has also been reported as one of the important risk factors for SSI [9]. There has been no reported significant correlation between biological reconstruction and SSI; however, high infection rate had been reported after biological reconstruction using an allograft or tumor-bearing bone graft [8]. Therefore, factors such as chemotherapy, radiation therapy, long operative time, and intraoperative blood loss, should be included in the multivariate analyses to evaluate efficacy of preventive technology. As aforementioned, several factors can be considered as risk factors of SSI.

A new preventive technique is needed to improve the outcome of bone tumor surgery in patients with risk factors for SSI. There are reports of new techniques to prevent postoperative infections that suggest, that silver coating and iodine coating can significantly prevent SSI [4, 32, 33]. This retrospective study regarding the efficacy of prophylactic coating on SSI is limited by the influence of various risk factors, including surgical site and operative time. As iodine-coated implants have been used to prevent SSI in patients with risk factors, such as chemotherapy and biological reconstruction, no significant preventive effect of an iodine-coated implant was identified by univariate analysis. However, results of multivariate analysis indicated that an iodine-coated implant significantly decreased the rate of SSI.

In the present study, there are several limitations including small number of patients with endoprosthesis, long period, and heterogenous group of tumor types, locations and reconstruction type. Basically, iodine-coated implants were used in patients with high risk of infection. In patients with malignant bone tumors, postoperative deep infection after endoprosthesis is major problem. In the present study, however, the number of patients with endoprostheses was small because tumor-bearing frozen or pasteurized bone graft using plate or intramedullary nail are popular procedure in Asian countries and performed in large part of the study patients. Furthermore, this retrospective study includes a heterogeneous patient population (tumor histology and type of implants). A prospective study with a suitable control group and a focus on tumor endoprostheses might be useful to investigate the efficacy of iodine-coated implants for reducing the incidence of deep infection after bone tumor resection. Reconstruction using endoprostheses has been thought to be a risk factor for deep infection. Iodine coating might be a promising technique for preventing postoperative deep infection in malignant bone tumor operations that call for implants as part of the reconstructions, but more study with larger numbers of patients is needed to confirm the advantages to use iodine-coated implants. In particular, future studies might focus specifically on endoprosthetic reconstructions, where the morbidity associated with infection is so very severe. If there is a lessening of infections with this technology, it may contribute to prevention of the development of multidrug-resistant bacteria resulting from long-term use of antibiotics and additional surgeries, which could reduce the cost of medical care, although future studies are needed to test this hypothesis.

## Conclusions

Our data indicate that pelvic tumor and long operative time are risk factors for SSI after malignant bone tumor resection and reconstruction. Iodine coating may be a promising technique for preventing SSI, although the present study has several limitations including decision making process for implantation of iodine-coated device, study period, and heterogenous group of tumor types, locations and reconstruction type. The effect of iodine-coating should be tested in prospective study to assess the efficacy of the technique.

## Abbreviations

CI: confidence interval
EWSR1: Ewing sarcoma breakpoint region 1
NNIS: The National Nosocomial Infections Surveillance
OR: odds ratio
SSI: surgical site infection
